# Middle Meningeal Artery Embolization for Chronic Subdural Hematoma Related to Autosomal Dominant Polycystic Kidney Disease

**DOI:** 10.7759/cureus.34970

**Published:** 2023-02-14

**Authors:** Hayes B Fountain, Bernardo A de Monaco, Jonathan Jagid, Ronald Benveniste, Joacir G Cordeiro

**Affiliations:** 1 Department of Neurological Surgery, University of Miami Miller School of Medicine, Miami, USA; 2 Neurosurgery, University of Sao Paulo, Sao Paulo, BRA

**Keywords:** mma, middle meningeal artery, embolization, adpkd, autosomal dominant polycystic kidney disease, bilateral subdural hematoma, chronic subdural hematoma, sdh, neurosurgery

## Abstract

Autosomal dominant polycystic kidney disease (ADPKD) is a connective tissue disease with vascular abnormalities involving multiple organs. The prevalence of ADPKD associated with a spontaneous subdural hematoma (SDH) is very low, with less than 10 cases reported in the literature to date. Symptomatic chronic SDH is classically treated with a twist drill, burr holes, or craniotomy. Recently, middle meningeal artery (MMA) embolization has emerged as an ancillary modality. We present the first case in the literature of a bilateral SDH in a young ADPKD patient successfully managed with MMA embolization. Moreover, we discuss the role of different treatment modalities on this subset of patients.

## Introduction

Autosomal dominant polycystic kidney disease (ADPKD) is a disease characterized by the involvement of multiple organs, frequently associated with vascular abnormalities [1]. Neurologic symptoms in patients with ADPKD are often related to intracranial aneurysms that generally manifest acutely as subarachnoid hemorrhage or transient ischemic attacks [2]. A small number of case reports have shown patients with ADPKD that presented with spontaneous subdural hematoma (SDH) manifesting as headache or hemiparesis [3]. The reported cases of SDH in ADPKD patients are unilateral and often associated with the imaging finding of arachnoid cysts [3-5]. We identified a single case report of an ADPKD patient with bilateral SDH and no arachnoid cysts in the literature [3]. Our case report presents the second case of spontaneous bilateral SDH in the setting of ADPKD without associated arachnoid cysts or vascular abnormalities on brain angiography.

Treatment options for SDH have classically been conservative or surgical. Conservative management may entail neuroexam monitoring, image surveillance, corticosteroids, and symptomatic therapy. Surgical intervention is typically employed in case of neurologic symptoms with significant signs of brain compression such as an image with over 5 mm of midline shift and/or hematoma thickness over 10 mm [6,7]. Endovascular treatment has recently emerged as an ancillary modality to treat chronic SDH (cSDH). It is performed with the embolization of the ipsilateral middle meningeal artery (MMA) [6]. The endovascular treatment aims at reducing the cSDH meningeal vascular supply which is believed to contribute to the chronic inflammatory process that culminates with the hematoma capsule formation. We present, to the best of our knowledge, the first case of bilateral SDH in the setting of ADPKD treated with MMA embolization associated with radiographic resolution of the chronic hematoma.

## Case presentation

**Figure 1:**
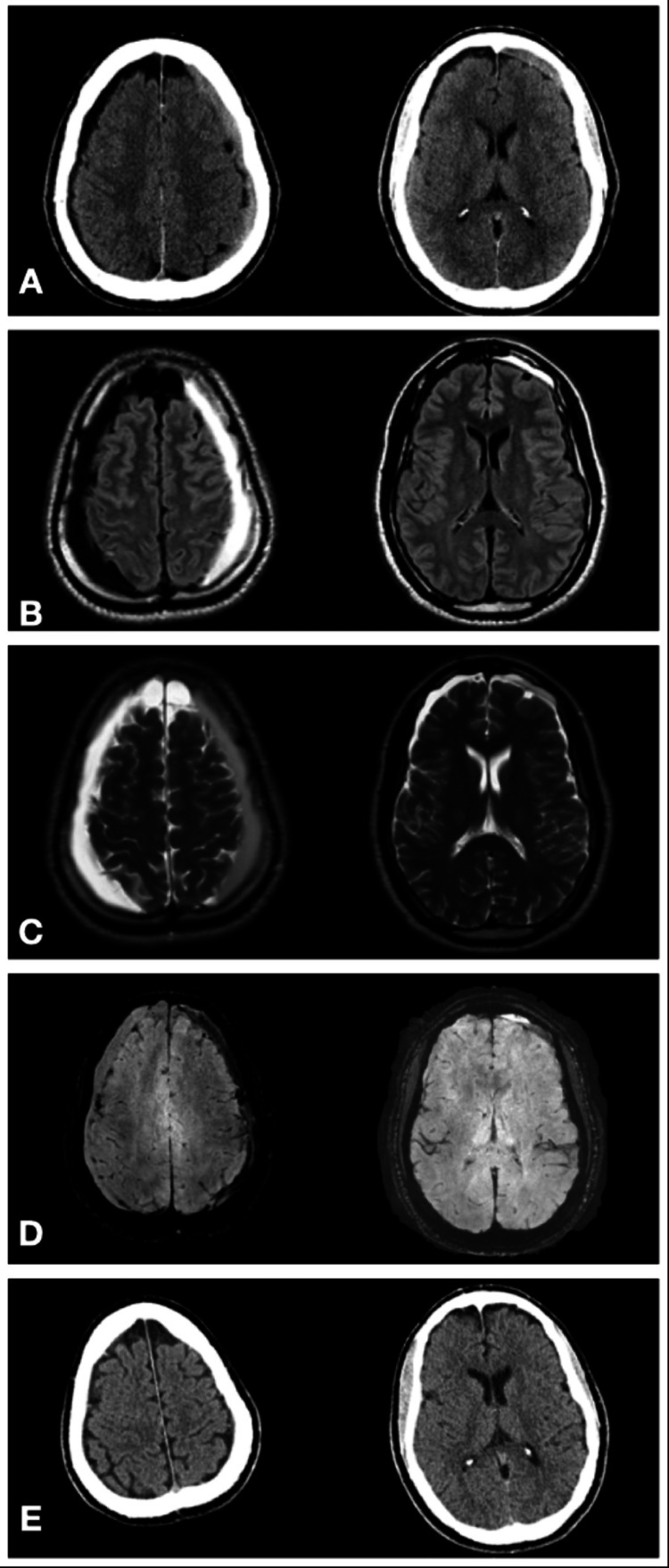
Panel A: CT at admission showing bilateral subdural hematomas, Panel B: T1 MRI at admission, Panel C: T2 MRI at admission, Panel D: SWI MRI at admission, and Panel E: CT at three-month follow-up after middle meningeal artery embolization showing resolution of bilateral subdural hematomas SWI: susceptibility-weighted imaging

**Figure 2:**
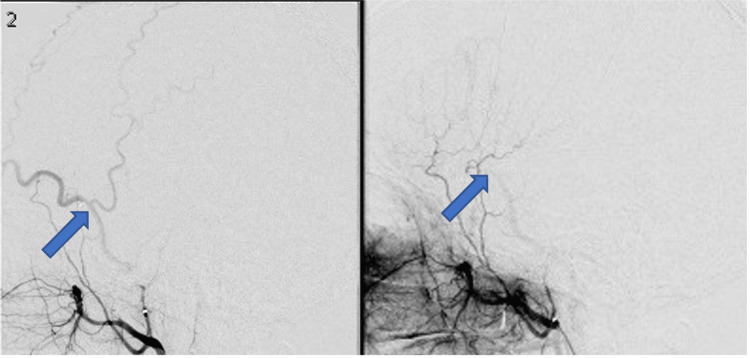
Diagnostic angiography showing the right middle meningeal artery bifurcation into the frontal and parietal branches prior to its embolization (arrow, left picture) and post embolization (arrow, right picture)

A 47-year-old African American male presented to the emergency room reporting one month of severe headaches that fluctuated in intensity and were not relieved by acetaminophen. He denied a history of trauma, syncope, seizure, or alcohol use. Past medical history of only hypertension managed with hydrochlorothiazide. His neurological exam was intact throughout. Subsequent head CT and MRI revealed acute hemispheric SDH (aSDH) on the left side and a chronic SDH on the right with no signs of brain atrophy (Figures 1A-1D). Brain computed tomography angiography (CTA) was unremarkable. During this initial admission, the patient was noted to have an elevated creatinine of 1.4 mg/dL, blood urea nitrogen (BUN) of 20 mg/dL, and estimated glomerular filtration rate (eGFR) of 65, signs indicating possible renal impairment. Abdominal ultrasound and MRI revealed multiple renal cysts consistent with ADPKD. Family history was negative for ADPKD or other renal diseases. MRI of the cervical, thoracic, and lumbar spine showed no evidence of a cerebrospinal fluid leak, arachnoid cysts, or other abnormalities. Hence, a diagnostic angiogram via radial artery was performed and the result was unremarkable. In the same session, a right-sided MMA embolization was accomplished. The frontal and parietal branches of the right MMA were visualized (Figure 2) and embolized using 1 cc of a 1:6 mixture of N-butyl cyanoacrylate (NBCA): lipiodol. Post-procedure angiogram confirmed successful embolization of the aforementioned MMA branches. The patient was given preoperative and postoperative IV fluids to avoid worsening his renal impairment. There were no post-procedural complications. The patient was discharged to home two days later. In the first outpatient follow-up, a week after discharge, the patient reported progressive headaches improvement. The repeat head CT during this visit showed the cSDH stable (right side) and the aSDH in the process of resorption (left side). At the three-month follow-up, he was asymptomatic and the head CT showed complete resolution of both hematomas (Figure 1E).

## Discussion

To the best of our knowledge, this is the first reported case of bilateral SDH in the setting of ADPKD treated with MMA embolization. This approach was associated with the complete resolution of the chronic hematoma. The contralateral acute SDH was managed conservatively and did not chronify.

The diagnosis of ADPKD is typically confirmed primarily by imaging [8]. In the absence of a family history of ADPKD, bilateral renal enlargement and the presence of cysts with the absence of other manifestations suggesting a different renal cystic disease provide sufficient evidence for the diagnosis of ADPKD [8]. Our patient met those criteria. He presented bilateral renal enlargement and innumerable cysts on a kidney ultrasound and MRI. Additionally, in up to 25% of ADPKD patients, no other family member is identified to share the condition [9]. In those cases, usually, the affected members either passed away or have a mild form of the disease. In around 5% of cases, the disease may be due to a new mutation or mosaicism [10]. For this reason, the diagnosis of ADPKD in our patient is supported despite the negative family history.

The exact mechanisms that lead to a subdural hematoma to chronify in the setting of ADPKD are yet to be determined. The likelihood of a subdural hemorrhage to become a cSDH increases with age, being around 56% in patients over 70 years old [11]. Hematoma thickness, use of anticoagulation, antiplatelets, and thrombocytopenia are also risk factors for chronification [11]. Our patient presented with a cSDH and contralateral acute SDH. It is unknown why the acute SDH did not chronify in the same manner as the contralateral cSDH. Potentially, the patient’s young age and lack of anticoagulation may have contributed. For this reason, we see the role of further research investigating the dynamics involved in SDH in ADPKD.

In 2020, Ng et al. published a randomized controlled trial with 41 patients comparing surgery with and without MMA embolization in patients with chronic SDH [12]. One recurrence of chronic SDH was reported in each group. Interestingly, patients who underwent MMA embolization and surgery had a statistically significantly higher hematoma reabsorption rate at three months post-procedure than those who received surgical treatment alone [12]. Our case adds to the growing reports of successful treatment of chronic SDH with MMA embolization alone but larger randomized control trials with longer follow-up times are needed to establish the efficacy of MMA embolization for chronic SDH [6]. We acknowledge that by their observational nature, case reports carry a limitation. It is possible that the chronic SDH in our case could have regressed without the MMA embolization as some non-ADPKD-related chronic SDH are known to do.

Reports of unilateral SDH in ADPKD patients are more common than bilateral SDH [3,4]. In 2000, Wijdicks et al. reported a single case of bilateral SDH in ADPKD while all the others were unilateral, totaling five cases [3]. In 2001, Holthouse et al. reported one ADPKD patient with unilateral SDH on presentation who later developed a contralateral SDH [4].

Chronic SDH is mostly seen in the elderly population typically associated with a significant degree of brain atrophy [7]. The patient in this case provides an atypical presentation being outside the usual age for spontaneous chronic SDH [7]. Additionally, this patient has an insidious presentation with a one-month history of progressive headaches without any history of trauma, syncope, or alcohol use, events that could potentially be an inciting traumatic event leading to SDH.

Roughly 70% of acute SDH are secondary to trauma. Less commonly, it may be associated with other medical conditions or spontaneous [13]. Non-traumatic causes include ruptured intracranial aneurysms, arteriovenous malformations, neoplasms, hypertensive cerebral hemorrhage, hematologic disorders, anticoagulant or thrombolytic therapy, cerebral amyloid angiopathy, dural arteriovenous fistula, and acquired immune deficiency syndrome [13]. Typically, patients with non-traumatic acute SDH present with abnormal MRI or abnormal vascular imaging, such as cerebral angiography or angiotomography pointing to the hemorrhage etiology. As our patient did not have any abnormal findings, we broadened the investigation until a potential underlying condition was identified.

An important consideration in ADPKD patients presenting with SDH who are treated with MMA embolization is that a common consequence of ADPKD is renal impairment. Renal impairment may be a relative contraindication to contrast administration for angiography in patients with sufficiently impaired kidney function (estimated glomerular filtration rate <30 mL/min/1.73 m^2^) [14]. In patients with a reduced estimated glomerular filtration rate, prophylaxis with normal saline is indicated. Our patient was given preoperative and postoperative IV fluids to avoid worsening his renal impairment. In our patient, contrast administration did not present any problems.

Notably, the patient had medically treated hypertension, likely secondary to the previously undiagnosed ADPKD. In ADPKD, the cysts that occupy the kidney volume are both direct and indirect causes of hypertension. These cysts directly interfere with tubular function in the kidney, which leads to fluid retention and hypertension. Additionally, it has been hypothesized that these cysts also cause the release of circulating renin leading to increased salt and water reabsorption and furthering hypertension [15]. These two pathological processes lead to increased pressure on the vessel walls, likely leading to microcirculation changes in the short term. Over the long term, this pathological hypertension may activate the inflammatory cascade, as the vessels remodel to accommodate the shearing forces from prolonged hypertension, leading to vessel fragility and leakage. The presence of these pathologic renal changes so early in life in ADPKD, which leads to such uniquely longstanding hypertension, may contribute to the risk that ADPKD portends to cause vessel fragility and leakage. This long-standing hypertension due to ADPKD may be a contributing factor in the development of SDH in ADPKD patients via microvascular changes, inflammatory cascade activation, and vessel remodeling and fragility. It is speculated that the subdural vessels of patients with ADPKD may be more susceptible to the effects of chronic hypertension than the general population. This aspect reinforces the importance of strict blood pressure control in ADPKD. A deep understanding of the underlying mechanisms involved in this association is yet to be achieved [3,5,7].

## Conclusions

In summary, we highlight the importance of considering SDH and other space-occupying lesions in the differential diagnosis of all adult patients with unexplained headaches, regardless of age. Likewise, ADPKD is a differential diagnosis to be considered in younger patients with spontaneous acute or chronic SDH. Our case provides further support for the accepted practice that acute SDH might be managed conservatively in neurologically stable patients. Moreover, there could be a role for endovascular treatment in chronic SDH cases that don’t require urgent surgical decompression or in postoperative recurrence. More investigation is required to better understand the underlying mechanisms involved in the generation and chronification of SDH in ADPKD.
